# Editorial: Insights in genetics, genomics and epigenomics of aging: 2025

**DOI:** 10.3389/fragi.2026.1786864

**Published:** 2026-02-25

**Authors:** Marcella Reale

**Affiliations:** Department of Innovative Technologies in Medicine and Dentistry, University “G. D’Annunzio”, Chieti, Italy

**Keywords:** aging, APOE, epigenetic, genetic, microbiome

The Research Topic “*Insights in Genetics, Genomics and Epigenomics of Aging*: *2025*” synthesizes recent advances and future perspectives of genetic, epigenetic, and environmental factors that affect the aging process profoundly. Aging is the consequence of a loss of homeostatic biological equilibrium, which drives biological decay in developed tissues. Studying the causes that can accelerate biological decay and measuring how far various biological elements have deviated from a youthful state may help to hypothesize individual’s health and predict biological age.

*Phua T*, in his manuscript emphasized what Palmer, (2022) said, namely, that aging is a process organized and expectable managed by intrinsic biological mechanisms indicated by assessable markers, and not only an echo of chronological age. And, in accord with (Dai and Shen, 2022) suggest that the “top-down” methodologies, influenced by OMICS, integrated with “bottom-up” strategies facilitate biological system investigations. Phua T suggests that between the ages of 40 and 60, the human biological clock begins to tick, and that precision health strategies and precision geromedicine for midlife are therefore necessary. This can reduce the incidence of chronic diseases, resulting in lower healthcare costs and greater sustainability within healthcare systems. The application of integrative precision geromedicine for midlife can be applied, for example, to age-related prostatic degeneration. The holistic dimension of integrative medicine suggests a dual medical approach, addressing age-related NO deficiency playing a crucial role in micro-angiopathy and tissue hypoperfusion, along with complementary therapies such as regular prostate drainage/massage to eliminate starch-like waste products, thus mitigating prostate size, pain, and inflammation related to compression.

The skin microbiota, profoundly affected by aging, influenced key skin functions as acidification, antimicrobial defense, lipid synthesis, and barrier integrity. As the skin microbiome is a pivotal component of skin health, their interaction is critical for maintaining the delicate balance between health and dermatological conditions, and the evaluation of age-dependent skin microbiome variations is essential. In fact, reduced epidermal thickness, slowed cell turnover, decreased collagen production, and changes in immune function, observed in aged skin as well as extrinsic factors such as sun exposure, pollutants, and smoking induce significant alterations in the microbiome. The study of Swaney et al. evaluate the differences in composition, species interactions, and predicted metabolic function of the bacterial community during aging across two distinct skin sites. Collectively, this work highlight how, a significant, site-specific and age-related shifts in the microbiome is dependent on where we are in lifespan, and a loss of microbiome robustness and a shift towards a hyperdiversified, fragile microbial community in old age. The authors suggest that disruptions to this dynamic microbial balance can influence risk for dermatological diseases as well as impact lifelong health, and that the evaluation of microbial community diversity coud be a key differentiating biomarkers of the skin microbiome across the lifespan.

APOE is known to be genetically associated with human longevity and Alzheimer’s disease, and the genetic polymorphisms of APOE have been widely related to human longevity and several age-related diseases. Many studies have been conducted to understand the mechanism of APOE in determining aging and to indicate potential therapeutic strategies that could be exploited to alleviate aging-related disorders, but the mechanistic role of APOE in aging is not yet fully understood.

The study of Holmes et al. fits into this context investigating how APOE4, changes in body weight and longevity are related. The role of aging in weight changes in the connection with APOE4 and longevity was explored using the causal mediation analysis (CMA) approach to uncover the mechanisms of genetic associations. A careful interpretation of these results can guide decision-makers when devising or refining interventions or policies that affect patient health. The results showed that APOE4 carriers have lower chances of survival to age 85+ were about 19%–22% more likely to die before age 85. The estimates suggest that 14%–19% of this association may be related to reaching the maximum weight at earlier ages and decline faster afterward compared to non-carriers. Postponing and attenuating the decline in weight in APOE4 carriers could be a promising target of pro-longevity interventions.

The original research by Bednorz et al., using unsupervised machine learning, investigated the influence of APOE and PICALM genotypes on cognitive performance. Analysis of cognitive and genetic information on the APOE and PICALM genes and demographic data revealed that the effects of the APOE ε3ε4 allele and the PICALM GG variant on cognitive functioning may vary by sex. The APOE ε3ε4 genotype was associated with poorer recognition in females with lower cognitive performance, while the APOE × PICALM combination was associated with poorer episodic memory in males. While the authors emphasize that the interpretation of these current findings must take into account the exploratory nature of this study and its focus on genetic influences on cognitive functioning and the performance is within the normative range, they suggest that these subtle differences may indicate early risk and warrant longitudinal monitoring.

In conclusion, I’d like to thank all the authors for their valuable contributions to the Research Topic “*Insights into Genetics, Genomics and Epigenomics of Aging: 2025*,” which highlights a growing interdisciplinary effort to assess the interplay between somatic mutations, epigenetic regulatory mutations, and epigenomic alterations during aging, and highlights how the use of omics technologies can provide valuable insights into the mechanisms underlying aging, offering potential interventions to promote healthy aging and combat age-related diseases.

As Research Topic editor I’d like to thank all reviewers for their valuable comments and suggestions that helped to improve the quality of the manuscripts.
